# Diagnosis of Leukaemia in Blood Slides Based on a Fine-Tuned and Highly Generalisable Deep Learning Model

**DOI:** 10.3390/s21092989

**Published:** 2021-04-24

**Authors:** Luis Vogado, Rodrigo Veras, Kelson Aires, Flávio Araújo, Romuere Silva, Moacir Ponti, João Manuel R. S. Tavares

**Affiliations:** 1 Departamento de Computação, Universidade Federal do Piauí, Teresina 64049-550, Brazil; lhvogado@gmail.com (L.V.); rveras@ufpi.edu.br (R.V.); kelson@ufpi.edu.br (K.A.); 2 Curso de Bacharelado em Sistemas de Informação, Universidade Federal do Piauí, Picos 64607-670, Brazil; flavio86@ufpi.edu.br (F.A.); romuere@ufpi.edu.br (R.S.); 3 Instituto de Ciências Matemáticas de de Computação, Universidade de São Paulo, São Carlos 13566-590, Brazil; ponti@usp.br; 4 Departamento de Engenharia Mecânica, Faculdade de Engenharia, Instituto de Ciência e Inovação em Engenharia Mecânica e Engenharia Industrial, Universidade do Porto, 4200-465 Porto, Portugal

**Keywords:** leukaemia classification, blood smear images, fine-tuning, CNN

## Abstract

Leukaemia is a dysfunction that affects the production of white blood cells in the bone marrow. Young cells are abnormally produced, replacing normal blood cells. Consequently, the person suffers problems in transporting oxygen and in fighting infections. This article proposes a convolutional neural network (CNN) named LeukNet that was inspired on convolutional blocks of VGG-16, but with smaller dense layers. To define the LeukNet parameters, we evaluated different CNNs models and fine-tuning methods using 18 image datasets, with different resolution, contrast, colour and texture characteristics. We applied data augmentation operations to expand the training dataset, and the 5-fold cross-validation led to an accuracy of 98.61%. To evaluate the CNNs generalisation ability, we applied a cross-dataset validation technique. The obtained accuracies using cross-dataset experiments on three datasets were 97.04, 82.46 and 70.24%, which overcome the accuracies obtained by current state-of-the-art methods. We conclude that using the most common and deepest CNNs may not be the best choice for applications where the images to be classified differ from those used in pre-training. Additionally, the adopted cross-dataset validation approach proved to be an excellent choice to evaluate the generalisation capability of a model, as it considers the model performance on unseen data, which is paramount for CAD systems.

## 1. Introduction

**Figure 1:**
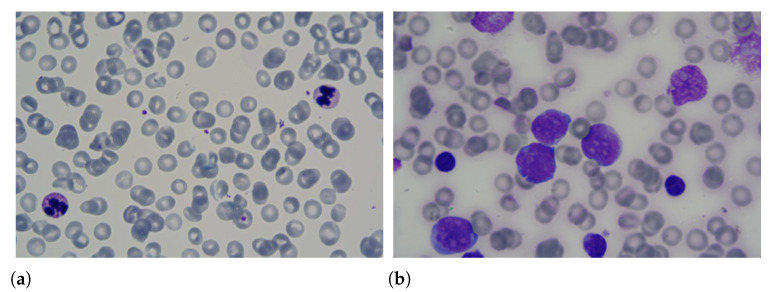
Samples of blood smears with (**a**) healthy and (**b**) unhealthy leukocytes.

Leukaemia is one of the most dangerous diseases according to the American Cancer Society (https://cancerstatisticscenter.cancer.org/#!/cancer-site/Leukemia (accessed on 1 March 2021)), with an estimate of 61,780 new cases and 22,840 deaths in 2019. This disease has an unknown cause and affects the production of white blood cells in the bone marrow. Due to the illness, young cells or blasts are produced abnormally, replacing healthy blood cells, i.e., white blood cells, red blood cells and platelets. Consequently, the affected person suffers from oxygen transport problems and infections. Among the forms of diagnosis of leukaemia are the lumbar puncture, myelogram, blood count and flow cytometry. Samples of blood smears with healthy and unhealthy leukocytes are shown in Figure 1.

Computer-aided diagnosis (CAD) systems aim to assist medical specialists by offering information that help them on their diagnosis [1]. Usually, such systems apply image processing and machine learning techniques to output diagnosis information, such as classification into “healthy” or “unhealthy”, and “benign” or “malignant”. They use annotated image tests, blood tests, biopsy results or other methods that are often available in datasets of known examples as input. Those systems are often employed to screen for diseases, providing the first diagnosis or offering a second opinion based on previously labelled examples [2].

One of the main issues in recent studies addressing medical imaging applications is the lack of heterogeneity in the image datasets that are used to evaluate the methods [3]. The used image datasets are often acquired using similar equipment, sampled from particular populations and annotated by a limited group of specialists. Evaluation based on holdout or cross-validation may be adequate to validate the performance of a method within a dataset, but it is unclear how the method generalises for other datasets. Considering deep learning methods, this is even more relevant, as it is known that models with sufficiently large capacity may be able to specialise to the used training data and fail to generalise [4]. Although transfer learning methods were shown to be useful in many applications, there is a relevant interest in studying how to choose the proper architecture and training strategies that preserve the usefulness of built models within the same domain of application but with changes, for example, as to the source of images, sensor, viewpoint and acquisition setup [5]. Therefore, there is a gap in the literature about guidelines for designing and evaluating CAD systems that are consistent, robust and reliable to be used in clinical practice.

In this context, we propose LeukNet, which is based on a convolutional neural network (CNN) that uses transfer learning concepts selected according to an extensive study of architectures, advanced training strategies and an in-depth discussion of evaluation. Therefore, a modified deeply fine-tuning (mDFT) method was employed in the training of the proposed model. LeukNet was evaluated on 3536 images of blood smears belonging to different sources, including hospitals and other institutions. Each dataset includes images acquired under different conditions, dimensions, and characteristics of colour, contrast and texture. The experimental results indicated the need for an evaluation protocol using a leave-one-dataset-out cross-validation (LODOCV), where the test is carried in one dataset. Additionally, the remaining datasets are used in the training process. This procedure is performed until all datasets are tested individually. This ensures that the CNN is not trained with any image of the datasets to be tested.

The remainder of the article is organised as follows. A description of related works is given in Section 2 along with the contributions achieved with current work. In Section 3, the material and methods used are described, including the proposed LeukNet solution. In Section 4, the results and their discussion are presented. Finally, the conclusions and perspectives for future work are given in Section 5.

## 2. Related Works and Contributions

Related works are discussed in this section regarding the image descriptor employed, sample size, validation method and achieved accuracy.

### 2.1. Handcrafted Features

Handcrafted features that have been often used to diagnose leukaemia from images are based on colour, texture and shape information, such as in the works by Putzu et al. [6], Vincent et al. [7], Patel and Mishra [8] and Singhal et al. [9]. The main drawback that can be found in these studies is the small size of the image datasets used in the experiments: Putzu et al. [6] used a database with 267 images and extracted features of shape, colour and texture. They evaluated an SVM classifier with 10-fold cross-validation and obtained 93.63% of accuracy. In Vincent et al. [7], the feature extraction process consisted of combining characteristics extracted from the grey-level co-occurrence matrix (GLCM), fractal dimension [10] and geometric attributes obtained from the segmentation of the leukocytes and lymphoblasts. The authors reported an accuracy of 97.70% obtained using a multilayer perceptron classifier in 100 images from the ALL-IDB1 database. Patel and Mishra used handcrafted statistical features and evaluated a training/test holdout setting made of 27 images. Singhal et al. [9] used a dataset of 260 images, which were described using texture features and evaluated using *k*-fold cross-validation. In both studies, an SVM-based classifier was used, achieving accuracies of 93.75 and 93.80%, respectively.

### 2.2. Deep Learning Models

Deep learning models have been increasingly used for computer-aided medical diagnoses, such as for the diagnosis of cervical cancer [11], melanoma [12] and breast cancer [13]. The referred studies use CNNs due to their capacity of learning hierarchical representations, from more general features in the first convolutional layers to more semantic features in the last layers.

Deep learning-based systems in leukaemia diagnosis have obtained promising results in recent years. For example, Thanh et al. [14] describe a CNN architecture for the diagnosis of leukaemia, which is similar to AlexNet, with five convolutional layers and two dense (or fully connected) ones [15]. The authors used the ALL-IDB 1 dataset (with 108 images) and the following data augmentation operators: rotation, translation, blurring and histogram equalisation, resulting in 1188 instances. By dividing the used dataset into training (70%) and testing (30%), they achieved 96.6% accuracy.

Shafique et al. [16] employed a CNN to diagnose different subtypes of acute lymphoid leukaemia (ALL). With four convolutional and three dense layers, the pre-trained AlexNet model was fine-tuned with an augmented ALL-IDB 2 dataset with a total of 760 images. The authors also evaluated the use of different colour systems as the input on CNN. The proposed model obtained 99.50% of accuracy for diagnosis between normal and disease images and 96.06% for the diagnosis of leukaemia subtypes. Similarly, Rehman et al. [17] also proposed a CNN for ALL subtypes’ diagnosis. Based on the AlexNet architecture, the authors adjusted the last layer to classify the L1, L2 and L3 subtypes and healthy leukocytes. To validate their approach, the authors employed a holdout evaluation and compared the results with the ones found in the literature.

Despite reporting an accuracy of 97.78%, previous studies outperformed their results, such as the work proposed by Shafique et al. [16], which reached 99.50%. Loey et al. [18] proposed a methodology based on the AlexNet architecture with fine-tuning to classify normal and abnormal leukaemia slides. The authors used a database with 564 images, which, after data augmentation, resulted in 2820 images. They obtained 100% of accuracy using the proposed methodology; however, the two classes used in both training and prediction belong to different databases. We believe that this fact contributed to obtaining these excellent results.

Pansombut et al. [19] presented a CNN for diagnosing two ALL subtypes: pre-T and pre-B. These subtypes have particular characteristics, and the use of deep learning techniques allows for the creation of an automatic and effective system for their identification. The proposed CNN has three convolutional layers for feature extraction, two fully connected layers and a sigmoid classification layer with three output neurons. The authors validated their model using the holdout strategy, and the used dataset had 363 images. The model obtained an accuracy of 81.74% in the experiments.

Ahmed et al. [20] studied two classifying leukaemia problems. The first problem was the differentiation between images with leukaemia and without the presence of the disease. The second one was the subclassification of leukaemia in ALL, acute myeloid leukaemia (AML), chronic lymphocytic leukaemia (CLL) and chronic myeloid leukaemia (CML). Therefore, the authors proposed a CNN with only two convolutional layers, two max-pooling layers and two fully connected layers. Two databases—ALL-IDB and ASH [21], totalling 903 images, were used in the experiments. A data augmentation technique was applied to avoid overfitting, which led to an increase of eight times the original dataset size. For the first experiment, the accuracies obtained were 88.50 and 81.74% in the first and second problem, respectively.

### 2.3. Feature Extraction with CNNs

Feature extraction with CNNs has also been proposed in this area. For example, in the methodology proposed by Vogado et al. [22], a CNN was employed for feature extraction by considering the activation of the last fully connected layer of three CNNs. The authors analysed the extracted characteristics and, due to the high dimensionality of the attributes vector, they performed attributes selection using the gain ratio technique. The final size of the characteristic vector was empirically established considering a balance between accuracy and dimensionality. An SVM incorporating a radial basis function (RBF) with standard parameters was used to classify the extracted vector. The validation methodology used was the *k*-fold cross-validation.

In the work of Sahlol et al. [23], it is used deep features extracted from the VGG-19 architecture. The extracted characteristics were selected using the Salp Swarm Algorithm (SESSA) and classified with an SVM. The authors evaluated the methodology in two datasets: ALL-IDB 2, with 260 images and C-NMC, a competition database with 10,661 images. To validate the method, they used 5-fold cross-validation, resulting in 96.11% of accuracy in the first dataset and of 87.9% in the second one, respectively.

Of these studies, only Vogado et al. [22] used more than two datasets for evaluation. Moreover, it was possible to verify that the commonly used evaluation protocols are the holdout and *k*-fold cross-validation within the same dataset, and when using CNNs, holdout was the most used technique. Due to the relatively small size of the available image datasets, one might question the convergence of the used classifiers and their ability to generalise as the relationship between the number of instances used for training and the complexity of the built model falls short in such scenarios [24]. When using the deep learning paradigm, a commonly used approach is to choose the model that achieved better performance in large datasets [25]. However, this may not be the case when it comes to different domains and data from various sources, which may favour models with a more restricted bias, demanding further investigation [5]. In this context, the use of more datasets would allow for better evaluation of the systems and their robustness in considering different sources of images. Thus, this article contribution is twofold: (i) to propose a CNN architecture and training strategy for leukaemia diagnosis offering extensive evaluation and discussion of the achieved results, and (ii) a novel evaluation protocol using images from different public available sources.

## 3. Materials and Methods

Let $X=\left\{x_{1},x_{2},\ldots ,x_{n}\right\}$ be a set made of *n* labelled blood smear images and $Y=\left\{y_{1},y_{2},\ldots ,y_{n}\right\}$ be a set where $y_{i}$ is a label indicating the presence (positive) or absence (negative) of leukaemia. In this article, the design of a classification function, i.e., f:X→Y, is investigated with the objective of learning a model that excels in terms of generalisation for distinct image datasets. The classification function *f* is implemented via a deep convolutional neural network. Therefore, the goal was to learn from a set of datasets $D=\left\{1,2,\ldots ,k\right\}$ gather from different sources *k* and still be able to obtain a model that can efficiently classify unseen data.

An extensive study of architectures and training strategies was performed to design network *f* to be used. As a result, transfer learning from five pre-trained architectures and four fine-tuning techniques was employed. The impact of data augmentation in the classification problem under study was also investigated.

### 3.1. Image Datasets

One of the challenges in building a medical aid system is the ability to accurately diagnose from image datasets with distinct characteristics. To evaluate the generalisation capability of the proposed model, 18 public datasets were used, which were then divided into development and performance sets.

Through experimentation, we used the development set to define the ideal configuration of the proposed model. This set was made of 17 datasets, totalling 3415 images, which present heterogeneity in terms of colour, contrast, resolution and texture. Furthermore, each of these datasets has a different balance ratio between classes, which helps to evaluate the robustness of the proposed model.

The performance set is a novel dataset, acquired at the Federal University of Goiás (UFG) in Brazil, referred to herein as the UFG dataset (https://hematologia.farmacia.ufg.br (accessed on 1 March 2021)). This dataset has 121 images acquired using different microscopes, with distinct characteristics of colour, texture and contrast. This is the first article to report results using this image dataset.

From the datasets used in our experiments, only three datasets are class-balanced: UFG, ALL-IDB1 and ALL-IDB2 [26], as indicated in Table 1. Some of them have images with only one leukocyte per image, and others have multiple leukocytes per image. Only the UFG and Bloodline datasets have these two kinds of images.

**Figure 2:**
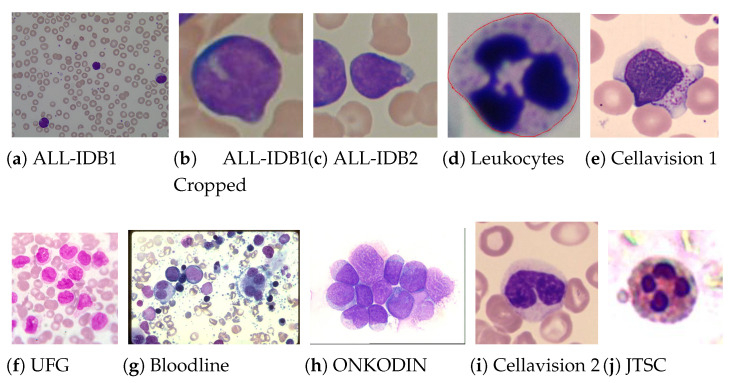
Examples of blood smear images from the used datasets, from which one can confirm the heterogeneity of the used images.

Samples from the 18 datasets, revealing distinct characteristics of colour, texture and contrast, and different original resolutions, are shown in Figure 2. All images are in the Red, Green and Blue (RGB) space and, due to the standard image input for ImageNet pre-trained CNN’s, were resized to 224×224 pixels. Although the image resizing leads to loss of spatial information in the images, our experiments showed that CNNs could find relevant features even in the reduced images, achieving good results as is shown later. The leukaemia images (http://www.leukemia-images.com/ (accessed on 1 March 2021)) and MIDB (http://www.midb.jp/blood_db/db.php?lang=en (accessed on 1 March 2021)) datasets were obtained from the indicated URLs.

### 3.2. Data Augmentation

Usually, CNNs have millions of parameters and need a large amount of data to be trained. Even to refine a small CNN, thousands of images are required. Faced with this challenge, state-of-the-art methods applied data augmentation techniques to overcome it [36].

Data augmentation consists of creating a new set of images using variations of the original images. The increase in data has the main goals of reducing the CNN overfitting and improving the generalisation of the trained model [37].

The image development set used in this work is relatively balanced: it contains 1001 non-pathological and 1182 pathological images. Therefore, data augmentation was applied equally in both classes.

**Figure 3:**
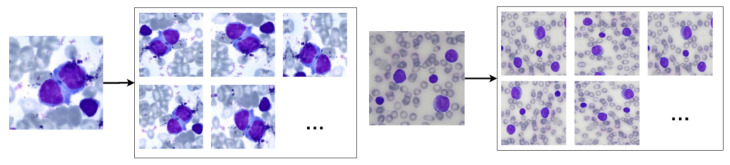
Examples of the results of the data augmentation operations when applied to non-pathological (**left**) and pathological (**right**) blood smear images.

Therefore, we used the random data augmentation technique provided by the Keras API. The chosen rotation interval was 40º, while the vertical, horizontal, shear and zoom translation interval was 0.2. We also used horizontal and vertical flip, as the nuclei images do not have asymmetry. The reflection fill technique was applied to replace black pixels resulting from the rotation and translation techniques. Finally, we normalised the image pixels to 0 (zero) and 1 (one). The augmentation resulted in an image dataset 20 times larger than the original one. Figure 3 shows results obtained by using these operations in blood smear images.

### 3.3. Transfer Learning

**Figure 4:**
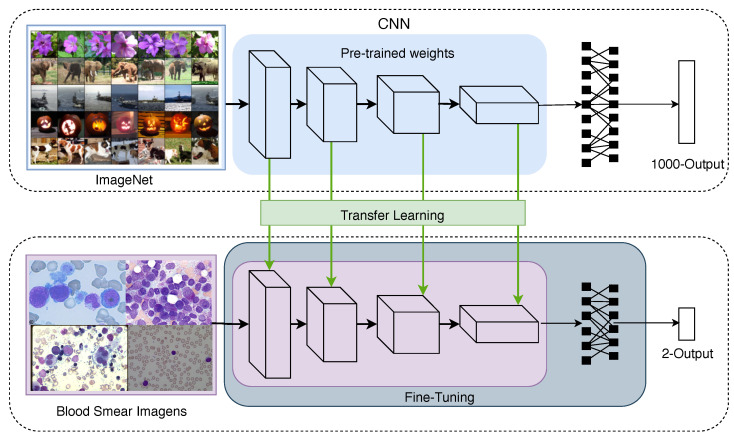
The transfer learning and fine-tuning techniques used in the development of the proposed CNN model.

The transfer learning technique that is often employed for convolutional networks uses weights that are pre-trained in large datasets, such as the ImageNet Challenge dataset [38]. This procedure decreases the requirement to retrain all parameters of the CNN from scratch [39]; Figure 4 depicts this idea. Note that some layers are usually copied from the pre-trained network, forming a base architecture, while other layers are randomly initialised and customised to the task at hand.

Two approaches are often employed when using pre-trained weights. One approach is to extract features as the activation maps of the pre-trained network layers, defining those as feature vectors to be used as input to shallow classifiers, such as an SVM [40]. The other one is to perform fine-tuning by creating a new classification layer. This approach has a higher computational cost than the first one, as it is necessary to resume the CNN training with the target dataset, adapting the desired model domain.

According to Tajbakhsh et al. [41] and Izadyyazdanabadi et al. [42], there are two types of fine-tuning: shallow fine-tuning (SFT) and deeply fine-tuning (DFT). SFT consists of freezing layers from the beginning of CNN, usually, the first convolutional layers, that are considered more general and allow representations of shape, texture and colour. The top layers are often domain-specific, carrying semantic content from the instance labels. Therefore, SFT provides greater specialisation in the later layers, while keeping the first ones.

The DFT approach allows training the entire network, adapting even the first layers. Although it has higher computational cost and requires larger amount of data, it can benefit applications where the target domain differs from the one used to pre-train the weights; for example, natural photographic images from the ImageNet dataset belonging to a very distinct domain relative to blood smear images.

**Figure 5:**
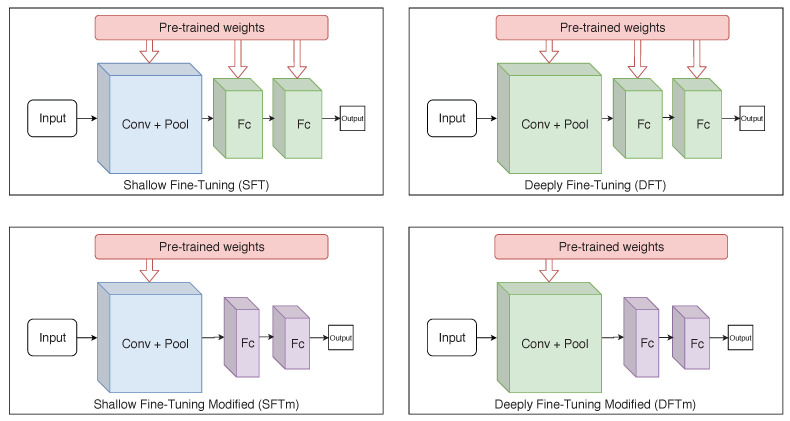
Simplified illustration of each used fine-tuning technique. (The blue colour, predominant in some layers, represents the freezing of parameters during the training, while the green colour represents that the layer is retrained. The purple colour exists only in the mSFT and mDFT techniques as the layers with these colours are not considered in the transfer learning and, therefore, their parameters are initially randomly defined).

As an alternative to the SFT and DFT approaches, additional experiments, referred to as modified shallow (mSFT) and deeply fine-tuning (mDFT), respectively, were developed here. In those experiments, dense layers—prior to the output layer—were replaced with new ones with smaller dimensionality (layers with 256, 512 and 1024 elements were evaluated). This decreases the network’s number of parameters, allowing faster training and making it less prone to overfitting. Figure 5 shows the operations of each fine-tuning technique used in this study. These experiments were performed because one can consider the used dataset as small. Note that previous studies reported that, for small datasets, smaller network architectures achieve better results; in particular, for binary classification and target domains that differ from those used for pre-training [5,43].

### 3.4. Evaluated Architectures

CNN architectures designed for the ImageNet Large Scale Visual Recognition Challenge (ILSVRC) [38] were explored in this study. Most of these CNN architectures are tailored to perform well in the ImageNet Challenge dataset, which has 1.4 million images containing 1000 categories of objects found in natural scenes. Indeed, the better a deep learning architecture performs on such a dataset, the better it transfers for other datasets of natural images, as verified by Kornblith et al. [25]. However, the same does not necessary happen for image datasets from other domains, such as from biomedical imaging, with fewer images for fine-tuning, as well as lower number of classes to classify, as it was demonstrated, for example, by Araujo et al. [43] and dos Santos et al. [44].

Thus, we choose sequential networks such as VGG-16 and VGG-19 [45], because these networks facilitate changes in the architecture structure, and residual and inception-based networks, as these networks presented better results in the ILSVRC. ResNet50 [46], InceptionV3 [47] and Xception [48] have fewer parameters than VGGNets, but have deeper architectures, as indicated in Table 2, where the architectures are presented in terms of year of publication, topological depth of the network (including batch normalisation and activation layers) and number of parameters.

In the mSFT and mDFT approaches, the initial size of the fully connected layers was based on Zang et al. [49], who studied cervical cancer images, which are similar to those for leukaemia diagnosis, and employed layers with dimensionality of 1024 and 256.

To evaluate the Inception architectures, the mSFT and mDFT approaches were used because when we fine-tuned InceptionV3, we added a new layer of global average pooling and a dense layer with 1024 elements with ReLu activation. For Xception, a dense layer with 128 elements was added. The output layer is the same as that presented in sequential architectures.

In the ResNet model, the same process as to InceptionV3 was performed. In the mSFT, only the added layers were trained, and the previous ones were frozen. On the other hand, in mDFT, all parameters were allowed to be fine-tuned.

### 3.5. Cross-Dataset Methodology

Fine-tuning was applied in the pre-trained CNN architectures to allow the model to classify images of blood slides. To evaluate the robustness of this architecture, experiments using all indicated datasets were performed. To evaluate classifiers, cross-validation is often used in different versions: *k*-fold, leave-one-out and holdout, where *k*-fold cross-validation is one of the method mostly used to validate CAD systems [6,9,22].

According to Diaz-Pinto et al. [50], CNNs take into account only the raw pixel information to classify images. Therefore, it is expected that the accuracy will be significantly affected when the model receives an image from a different dataset as input from those used in training or validation. This situation happens due to the changes in the images resulting from their origin and acquisition conditions. Therefore, methods that well classify images of a dataset will not necessarily succeed with images of other datasets. Thus, a critical procedure to evaluate the classifier performance is to use images obtained from distinct datasets.

We consider that *k*-fold cross-validation within a given dataset does not simulate a real scenario’s conditions because, when applying folding division, similar examples from the same dataset are likely to be present in both training and test sets. Furthermore, the use of *k*-fold cross-validation enables the classifier to be trained with examples from the same image dataset. Thus, leave-one-dataset-out cross-validation (LODOCV), a validation for systems that operate on several datasets from different sources, was employed un this study. Therefore, considering that the number of datasets available is *d*, d−1 datasets were used for training, and the method was evaluated on the unseen dataset. This procedure was repeated until all datasets were individually tested, which ensured that none of the images in a dataset were presented in both training and testing.

As shown in Table 1, 15 of the 18 datasets contained images from a single class. Thus, only the datasets with both classes’ images were used in the test step in LODOCV: ALL-IDB 1, ALL-IDB 2 and UFG datasets. For example, in the first experiment, ALL-IDB 1 was used as a test set, while the other 17 datasets were used in training. This process was repeated for ALL-IDB 2 and UFG datasets.

## 4. Experiments

The results obtained from the experiments performed were evaluated in terms of accuracy (A), precision (P), recall (R), specificity (S) and Matthews correlation coefficient (MCC) [51] (The used image databases and the developed codes are available at: https://git.io/JOCYu (accessed on 1 March 2021)). Because the new layers were trained from randomly initialised weighs, five runs were performed to compute mean and standard deviation of the evaluation metrics. The results were compared against those of the eight state-of-the-art methods, including standard feature extraction methods for colour, shape and texture [6], and CNN-based methodologies [22]. All experiments were carried out on a PC with a 3.6 GHz Intel^®^Xeon^™^processor, 24 GB of RAM, and a NVIDIA TITAN XP 12 GB graphics card.

### 4.1. Models and Fine-Tuning Evaluation

An ablation study was conducted to define the base architecture of the proposed model and the training methodology. Through validation by LODOCV, the development set was evaluated along the experiments using the ALL-IDB 1 and ALL-IDB 2 datasets. As already mentioned, this validation methodology was selected because it provides a more critical evaluation than *k*-fold cross-validation, simulating the actual training and testing conditions.

In the following experiments, the hyperparameters were empirically defined, following literature standards for training CNNs, and maintained constant in all experiments. The defined learning rate was 0.001, while the weight decay was 0.0001. The size of the mini-batch was defined as 32, and the binary cross-entropy was used as the loss function.

Table 3 presents the results obtained using the VGG-16 architecture. All fine-tuning approaches archived accuracy over 79% in the ALL-IDB1 dataset. However, among them, it was observed that the mDFT approach achieved the best results for both datasets.

During the training phase, it was verified that the lower loss does not always lead to the best accuracy, as well as the opposite. We obtained lower results in the ALL-IDB 2 dataset relative to the ALL-IDB 1 dataset. This ALL-IDB performance was due to the type of image classified: this dataset contained only one leukocyte per image, while ALL-IDB 1 has several. The presence of numerous leukocytes in the slide may denote the existence of the disease, thus facilitating its classification.

The results obtained by the VGG-19 architecture are presented in Table 4. From the data shown in this table, one can verify that DFT archived better accuracy, recall and MCC rates. However, mDFT, as in the VGG-16 case, obtained high rates compared with the other approaches, with 96.95% and 96.30% precision and specificity, respectively.

The results obtained using InceptionV3 and Xception are presented in Table 5 and Table 6, respectively. From these tables, one can realise that the mDFT technique was more effective than the mSFT technique. When comparing the accuracy obtained by the two architectures, Xception achieved better results in both datasets. However, when we compared those outcomes with the ones obtained using sequential architectures, there was a decrease in performance. Therefore, we concluded that this was because these architectures deal better with greater complexity in terms of the amount of data and classes than the other ones.

Finally, Table 7 presents the results obtained using ResNet50. This architecture did not originally have convolutional layers, so for fine-tuning, fully connected layers were added at the end of their structure. The achieved results were superior to the ones obtained by the Inception architecture. It is possible to observe that in the ALL-IDB 2 experiments, this architecture obtained an accuracy of 69.46% and a MCC of 40.65%, which are higher than the ones obtained by InceptionV3 and Xception. Similar to other architectures, the mSFT technique was still inferior to the mDFT. Therefore, we believe that both ResNet and other Inception-type architectures work best when fully retrained. Note that from the results obtained with mSFT with ALL-IDB1, it was possible to conclude that ResNet50 could not correctly generalise the classes, classifying all the examples in just one class. This caused a decrease in the average accuracy and recall, and a value of 0 (zero) as to the MCC metric.

LeukNet was designed after analysing the previously described results, where VGG-16 and VGG-19 architectures achieved the best outcomes, with similar values for the mDFT approach in the ALL-IDB2 dataset. Therefore, the Student’s *t*-test [52] was performed to statistically compare the results at a significance level of 5%. From the test performed, it was possible to conclude that the results were equivalent. Therefore, VGG-16 was selected due to its smaller number of trainable parameters.

According to Kornblith et al. [25], the best-performing architectures on ImageNet can provide better feature extraction and fine-tuning. However, the authors observed this fact only in photographic datasets. In datasets with fine-grained images, the effects of pre-training with ImageNet were considered small. The current study indicated that the features obtained from ImageNet are not adequately transferred to such datasets. According to Sipes et al. [53], leukaemia images are considered fine-grained images. This fact explains why the results achieved by VGG-16 and VGG-19 were superior to the ones obtained by the other CNNs.

Additionally, a running time analysis as to the CNNs training and classification of an image was conducted. Table 8 presents the results obtained for the evaluated architectures. This analysis was limited to refined models through mDFT because these models presented the best results in the classification.

Regarding the training time, the Inception V3 network was the fastest (32 min) and Xception the slowest (over 40 min). The running time to classify a single new image was in the order of 0.01 s or less for all the networks under comparison. In practice, all running times can be considered similar as a training under one hour and a classification under 0.01 s mean no restriction as to the practical application of the proposed methodology.

### 4.2. Proposed Model: LeukNet

The final LeukNet model uses a VGG-16 convolutional backbone, with new dense layers with lower dimensionality, and a training strategy based on transfer learning with mDFT. Experiments varying the size of the fully connected layers were also performed to find the best compromise between accuracy and loss (Table 9), which showed that the highest accuracy was achieved with 1024 and 256 neurons.

**Figure 6:**
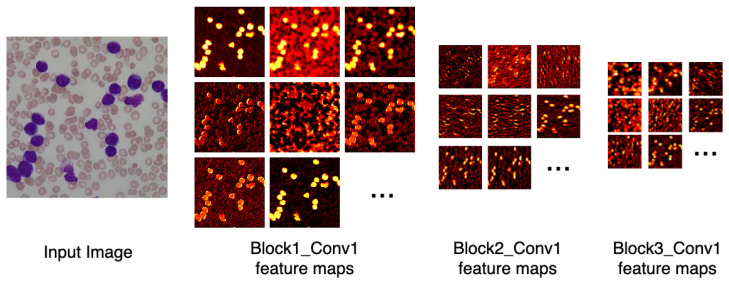
Heatmap of some LeukNet’s convolutional filters output.

Figure 6 depicts the output of some of LeukNet’s convolutional filters as heat maps. It can be seen in this figure that the CNN excludes the background and defines the cytoplasm and leukocyte nucleus as regions of interest. However, the nuclei region (regions in yellow tone in the figure) is considered to be the most crucial region for classification in the application addressed here.

To demonstrate the generalisation ability of the proposed model, a validation experiment was conducted using a random set containing 25% of the available images, and its accuracy and loss throughout the epochs were computed. Note that models tend to overfitting when they cannot generalise for a new set.

**Figure 7:**
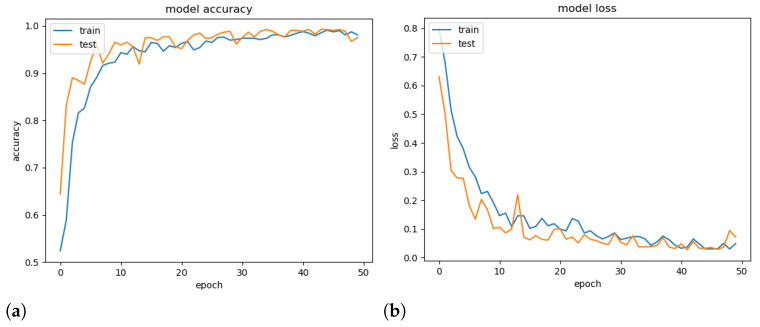
Training and validation accuracy (**a**) and training and validation loss (**b**) versus number of training epochs.

Figure 7 presents the obtained accuracy and loss ratio of the training and validation sets over the training epochs. One can observe that the results achieved in the validation set decrease with training, which characterises a good generalisation capacity [36]. From the results, it is possible to conclude that there was no overfitting during training. We attribute this fact to the decrease in complexity provided by the mDFT and data augmentation techniques.

**Figure 8:**
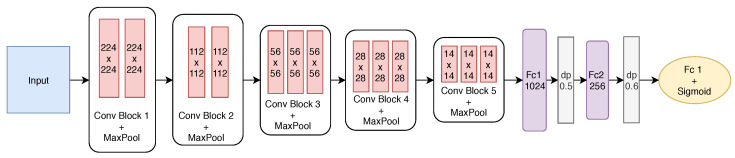
Detailed structure of the proposed CNN after the fine-tuning.

The best-built model has five convolutional blocks and two fully connected layers. After each convolutional block, max pooling is employed. The first two blocks have only two convolutional layers, while the remaining ones have three layers. The first block has 64 filters with size 3×3. From the second block on, the number of filters is doubled to 128, and after the convolution, the pooling operation reduces the filter size. Finally, the last two convolutional blocks have the same number of filters. Figure 8 shows the final structure of the proposed model.

To define the size of the two fully connected (FC) layers, the effect of the number of neurons was investigated, varying from 1024 to 256 at FC1 and FC2. To avoid overfitting, dropout (dp) was also employed after each fully connected layer with rates of 0.5 and 0.6, respectively. As we are dealing with a binary classification problem, the output layer has one neuron with the sigmoid activation function.

The stochastic gradient descent (SGD) optimisation algorithm was employed with a batch size of 32 and for a total of 50 epochs. Therefore, we used 0.001 and 0.8 for the learning rate and the momentum, respectively. The loss function used during fine-tuning was the binary cross-entropy to allow computing the gradients at each iteration.

**Figure 9:**
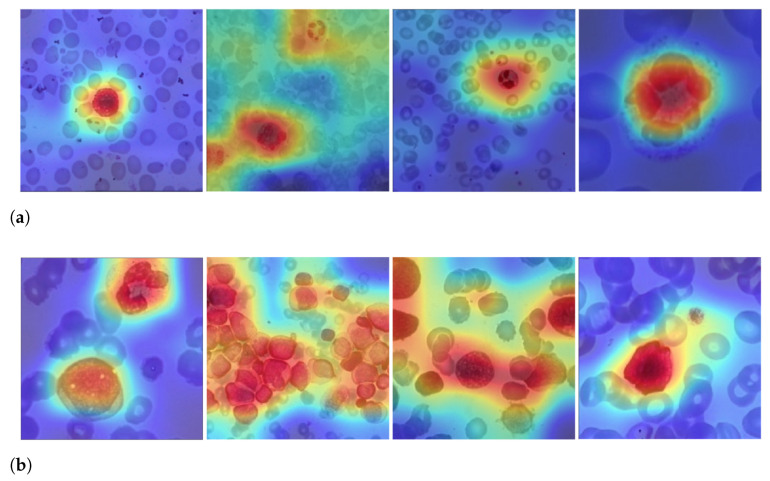
Examples of activation maps for blood slides of (**a**) healthy and (**b**) unhealthy images.

Figure 9 shows examples of LeukNet activation maps for the two classes under study. In this figure, it is possible to identify which regions are used to differentiate healthy images from those with leukemia. In the shown activation maps, blue tones mean low activation and indicate that the correspondent regions are of little importance for the final classification; in contrast, red tones are associated to the most critical regions for the final classification.

The number of leukocytes varies in the input images, causing LeukNet to generate different activation map patterns as shown in Figure 9. Furthermore, as it is trained using different datasets, the proposed model can adapt to different characteristics.

Interpretation is still a challenge in CNNs, but activation maps indicate that LeukNet gives more importance to regions containing disease patterns, as one can see, especially, in Figure 9a. From Figure 9, note that leukocytes and lymphoblasts are highlighted in the activation regions. Additionally, note that the number of leukocytes and their shape are considered essential aspects in detecting leukemia.

### 4.3. Beyond CNN Results with a Features Space Analysis

In order to go beyond the results obtained by fine-tuning the CNNs, we carried out two additional analyses using the features spaces formed by two models. In particular, the goal was to compare the models in terms of the linear separability of the feature spaces generated by the layer before the network classifier (output layer). Because we employed a linear SVM, which has strong learning guarantees, better results would favour models with better generalisation capabilities [24].

The analyses were performed according to two scenarios. The first scenario consisted of validation with LODOCV using feature extraction with pre-trained VGG-16 on ImageNet and fine-tuned VGG-16. The second one used databases tested in LODOCV (ALL-IDB 1 and ALL-IDB 2) individually as input for the *k*-fold cross-validation with a *k* value equal to 5. Both experiments used the same pre-trained models for feature extraction.

For the model pre-trained with ImageNet and those refined with DFT and SFT techniques, the output vector had 4096 features. Therefore, to analyse the intrinsic dimensionality in the data, a principal component analysis (PCA) was applied to reduce the vector to its 100 principal components. Table 10 presents the results obtained by the two performed analyses.

From the results in Table 10, it is possible to realise that in experiments with multiple datasets (LODOCV experiment), mDFT provided a superior linear separability of the data. However, for only one dataset (*k*-fold cross-validation experiment), the DFT showed better results. The advantage of mDFT in the first experiment was that it restricts dense layers in dimensionality (from 4096 in the original model to 256 in the proposed model), making the model robust to images from different datasets. The DFT uses a larger output, and it consequently has more “degrees of freedom” in the pre-trained model, which can cause overfitting in datasets used for fine-tuning, reducing the accuracy in an experiment with multiple datasets.

This analysis confirms previous findings which indicate that models with a more restricted bias, i.e., in terms of their space of admissible functions, may transfer better for different domains [4] in comparison to the same domain, which in the case of the widely used ImageNet dataset are mostly natural images and photographic data [25]. Furthermore, it is clear how a high *k*-fold cross-validation measure obtained by using an off-the-shelf CNN model, e.g., trained in ImageNet, is severely impacted when using a more realistic scenario concerning the different source and target datasets, which indicates the importance of transfer learning [54].

**Figure 10:**
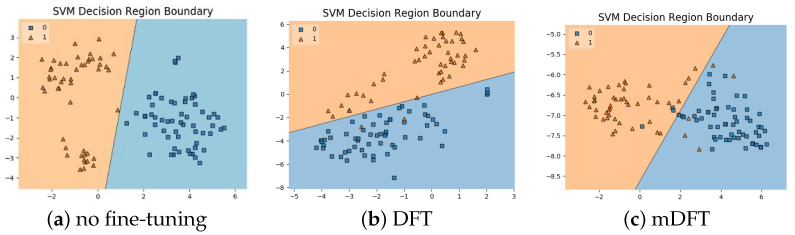
ALL-IDB 1 dataset visualisations using t-SNE projection in 2D along with the estimated decision boundaries using Linear SVM classifiers for different feature extraction methods: (**a**) no fine-tuning, (**b**) DFT and (**c**) mDFT.

**Figure 11:**
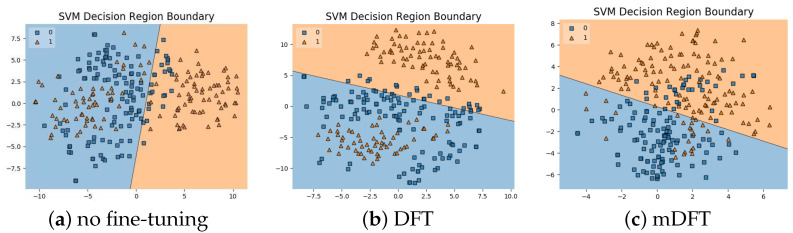
ALL-IDB 2 dataset visualisations using t-SNE projection in 2D along with the estimated decision boundaries using linear SVM classifiers for different feature extraction methods: (**a**) no fine-tuning, (**b**) DFT and (**c**) mDFT.

In addition to the classification experiments, we also visualised the feature spaces using a t-SNE projection, with the respective decision boundaries estimated to the 2D case, both for ALL-IDB 1, Figure 10, and ALL-IDB 2, Figure 11. From these figures, it is possible to note how the decision boundaries show good feature spaces with good discrimination capability. Furthermore, it is clear how ALL-IDB2 is a more challenging dataset, and that the mDFT tends to produce a space that better separates the classes compared to the greater classes overlap shown in DFT and spaces without fine-tuning, Figure 11.

**Figure 12:**
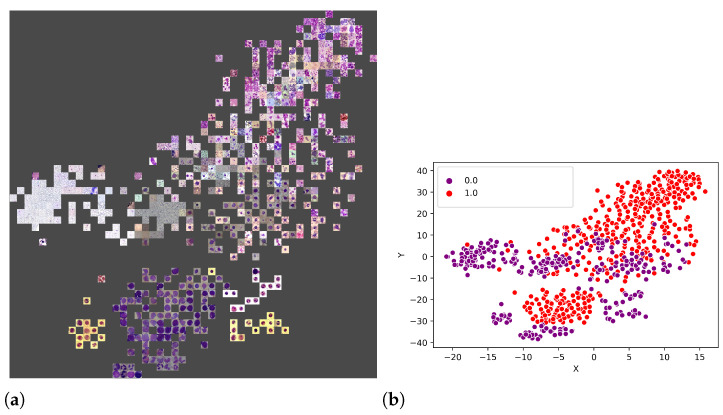
T-SNE visualisation with the represented points (**a**) and the division of classes (**b**) (0 = pathological and 1 = non-pathological).

An additional experiment was performed to better understand which features were used by the CNNs to separate the classes. Thus, from the union of the 18 datasets (totalling 3536 images), 80% of the images were randomly selected to form a training set, and the remaining 20% were used as the test set. Figure 11a illustrates the visual attributes that contributed to the classification using the t-SNE. Some of these attributes are the number of leukocytes per slide, the colour and zoom. However, in Figure 12b, one can also observe that it is impossible to separate the set linearly, requiring a more sophisticated prediction function, such as that provided by LeukNet.

### 4.4. Discussion

An interesting discussion in the classification and segmentation of medical pathology images is related to colour normalisation. In [55], the authors evaluated the influence of colour normalisation in the classification of lymphoma images and concluded that the best classification rate was obtained with features extracted from the images, i.e., without colour normalisation. Another study [56] evaluated the impact of colour normalisation in convolutional neural network-based nuclei segmentation in blood smear images, and it was concluded that, despite the colour variability in the original images, the used CNN model could effectively segment the nuclei presented in the original images. Thus, in this study, we chose not to apply colour normalisation strategies.

The results presented in Section 4.1 were obtained using the LODOCV strategy; however, other studies do not use this validation strategy. Thus, *k*-fold cross-validation, with *k* = 5 in all of the 3536 images from the available 18 datasets, was applied to compare the results of the proposed approach with the ones obtained by state-of-the-art methods. Table 11 presents the results achieved by the approaches under comparison; the indicated accuracy values for the state-of-the-art methods were gather from their original articles.

First, from Table 11, it is possible to verify that the number of images used in all competing methods is inferior to those presented in our experiments. Several authors used feature extraction techniques based on texture, shape and colour [6,7,8,9]. These methods achieved accuracies of 93.63, 97.7, 93.75 and 93.80%. The use of a single dataset and the accuracy obtained in the experiments proposed by these methods expose the lack of robustness compared with other state-of-the-art approaches.

Among the works based in deep learning techniques, Shafique et al. [16], Rehman et al. [17] and Pansombut et al. [19] proposed solutions for the classification of leukaemia subtypes. With the use of shallower and less complex CNNs, the authors were able to deal with small databases without compromising the CNN training as they did not use data augmentation techniques.

Thanh et al. [14], Ahmed et al. [20] and Loey et al. [18] tackled the classification between leukaemia and healthy images, as in this work. According to the results obtained by the proposed model using the image development set, it can be concluded that more complex architectures, i.e., with a higher number of parameters, produced better success rates in tests like LODOCV, and are more challenging than *k*-fold cross-validation and holdout. Among the previously mentioned studies, only Thanh et al. [14] presented an architecture with high complexity. However, in terms of architecture depth, LeukNet presents a more extensive set of convolutional filters, which allows the extraction of more feature maps.

Among the mentioned studies, Vogado et al. [22] presented experiments in more than two datasets: eight of the 14 that were used in this work. Among the best results, we observed that both Loey et al. [18] and Vogado et al. [22] obtained results superior to LeukNet. However, we must emphasise that Loey et al. [18] performed tests where the classes are represented by two homogeneous databases, which justifies the high accuracy obtained. Because Vogado et al. [22] was the only group to use the *k*-fold method for cross-validation, we compared our method with theirs in more detail below.

Table 12 presents the comparative result of the proposed method and the one of Vogado et al. [22]. In this experiment, we performed twenty *k*-fold cross-validation executions (*k* = 5) on 3415 images from 17 image datasets (the UFG dataset was separated for a second experiment). For comparison purposes, an experiment of training the VGG-16 from random weights is described.

Vogado et al. [22] used eight of the seventeen datasets used in this study. Comparing the results presented in Table 11 and Table 12, the competing method shows lower accuracy after inclusion of new images. In particular, the ASH, Bloodline and ONKODIN datasets are composed of images with distinct resolutions, textures and different colour characteristics. According to the results shown in Table 12, one can observe that using the Student’s *t*-test with a significance level of 5%, the results of VGG-16 and LeukNet can be considered equivalent, and both are superior to the competing method.

To demonstrate the generalisation capacity of LeukNet, Table 13 shows average results of applying the models generated by the previous experiment in an external dataset as a test set. The UFG set is a novel dataset, which was never used in previous studies, and is particularly challenging for three reasons: (1) the dataset is formed up of images acquired by different microscopes, and according to different resolutions and lighting conditions (2) has complete slide images and images with only one leukocyte, and (3) among all the datasets used in this study, the UFG dataset is the one with the highest diversity within the leukemia class, since it has examples of images with ALL, AML, CLL and CML subtypes.

From Table 13, one can conclude that the three approaches obtained lower results when compared to those obtained by the *k*-fold cross-validation. However, the decay of the proposed model was more moderate (from 98.61 to 70.24%) than that of VGG-16 (98.64 to 65.94%) and that in [22] (from 92.79 to 52.06%). This result suggests that LeukNet can generalise better than the methods in the literature. Thus, it can be concluded that this superior generalisation is due to the use of larger data and the precise definition of the convolutional neural network parameters as was conducted in this study.

## 5. Conclusions

This work presented a novel CNN architecture and training strategy to diagnose leukaemia in blood smear images. Several architectures, fine-tuning schemes and parameters were studied to define the proposed model. This allowed us to develop a model for diagnosis that is more precise and robust than the methods presented in current state-of-the-art works.

From the comparisons performed against previous studies, some conclusions may be drawn as to the computational leukaemia diagnosis from images. First, fine-tuning may be more efficient than off-the-shelf feature extraction. Second, CNNs with more representations through feature maps perform better in cross-dataset experiments. Furthermore, the choice of the fine-tuning technique is essential for the correct definition of CNN parameters. As for blood sample images belong to a different domain to those used to pre-train the layers, the adjusting of all of the layers is preferable.

The use of the LODOCV evaluation demonstrated the need for more challenging experiments towards a better generalisation capability, allowing a model to perform satisfactorily even on an unseen dataset. New studies are needed to investigate the feature representations learned by LeukNet, when compared to pre-trained models or even handcrafted features. Future work may also investigate the use of generative adversarial networks in increasing data availability; particularly, these networks can generate heterogeneous images that sufficiently represent the original distribution. Finally, the evaluation of the computational results by additional experts would be crucial for the routine use of the proposed model.

## Figures and Tables

**Table 1:** Summary of the image datasets used in the experiments.

Dataset	Non-Pathological	Pathological	Total	Ref.
ALL-IDB 1	59	49	108	[26]
ALL-IDB 1 (Crop)	0	510	510	[26]
ALL-IDB 2	130	130	260	[26]
Leukocytes	149	0	149	[27]
CellaVision	109	0	109	[28]
Atlas	0	88	88	-
Omid et al., 2014	154	0	154	[29]
Omid et al., 2015	0	27	27	[30]
ASH	0	96	96	[21]
Bloodline	0	204	204	[31]
ONKODIN	0	78	78	[32]
CellaVision 2	100	0	100	[33]
JTSC	300	0	300	[33]
UFG	57	64	121	-
PN-ALL Dataset	0	30	30	[34]
leukemia-images	0	140	140	link
MIDB Dataset	0	673	673	link
LISC Dataset	376	0	376	[35]
**Total**	**1434**	**2102**	**3536**	-

**Table 2:** Characteristics of the evaluated deep learning models.

Model	Topological Depth	Number of Parameters	Year
VGG-16	23	138,357,544	2014
VGG-19	26	143,667,240	2014
ResNet50	168	25,636,712	2015
InceptionV3	159	23,851,784	2016
Xception	126	22,910,480	2017

**Table 3:** Results obtained using the VGG-16 architecture according to 50 epochs (best values in bold).

Approach	A (%)	P (%)	R (%)	S (%)	MCC (%)
**ALL-IDB 1**					
SFT	79.07 ± 2.13	79.76 ± 7.95	74.29 ± 9.42	83.05 ± 9.28	58.52 ± 4.05
mSFT	81.67 ± 2.01	90.03 ± 8.37	68.16 ± 6.71	92.88 ± 6.93	64.41 ± 4.73
DFT	96.48 ± 1.21	95.97 ± 1.31	96.33 ± 3.35	96.61 ± 1.20	92.96 ± 2.44
mDFT	**97.04 ± 1.21**	**96.42 ± 2.45**	**97.14 ± 1.83**	**96.95 ± 2.21**	**94.07 ± 2.36**
**ALL-IDB 2**					
SFT	72.46 ± 2.15	72.68 ± 5.55	73.38 ± 7.33	71.54 ± 8.99	45.42 ± 4.69
mSFT	78.15 ± 0.01	76.49 ± 0.04	82.61 ± 0.09	**73.69 ± 0.08**	57.33 ± 2.77
DFT	66.08 ± 7.37	63.83 ± 7.42	75.85 ± 13.92	56.31 ± 14.24	33.80 ± 14.7
mDFT	**82.46 ± 0.02**	**77.59 ± 0.04**	**92.30 ± 0.08**	72.61 ± 0.09	**66.96 ± 4.96**

**Table 4:** Results obtained using the VGG-19 architecture according to 50 epochs (best values in bold).

Approach	A (%)	P (%)	R (%)	S (%)	MCC (%)
**ALL-IDB 1**					
SFT	87.78 ± 0.77	91.35 ± 4.74	81.22 ± 6.52	93.22 ± 4.32	75.84 ± 1.36
mSFT	81.11 ± 1.92	81.30 ± 6.72	77.55 ± 11.45	84.07 ± 8.00	62.70 ± 3.31
DFT	**97.04 ± 0.41**	95.98 ± 0.04	**97.55 ± 0.91**	96.61 ± 0.00	**94.04 ± 0.85**
mDFT	94.81 ± 1.40	**96.30 ± 2.37**	92.24 ± 4.87	**96.95 ± 2.21**	89.71 ± 2.69
**ALL-IDB 2**					
SFT	73.23 ± 2.22	74.27 ± 4.02	71.69 ± 5.26	**74.77 ± 6.54**	46.70 ± 4.44
mSFT	75.31 ± 1.37	72.83 ± 3.62	81.69 ± 8.35	68.92 ± 8.12	51.61 ± 3.07
DFT	75.77 ± 5.10	71.65 ± 5.95	**86.62 ± 8.24**	64.92 ± 10.40	53.33 ± 10.37
mDFT	**79.62 ± 6.31**	**77.54 ± 8.53**	85.38 ± 9.53	73.85 ± 12.93	**60.43 ± 12.61**

**Table 5:** Results obtained using the Inception V3 architecture according to 50 epochs (best values in bold).

Approach	A (%)	P (%)	R (%)	S (%)	MCC (%)
**ALL-IDB 1**					
mSFT	45.74 ± 7.90	42.29 ± 8.03	59.59 ± 26.15	34.24 ± 22.08	−8.37 ± 17.55
mDFT	**65.56 ± 9.79**	**58.47 ± 17.18**	**73.92 ± 16.99**	**60.91 ± 19.90**	**35.54 ± 15.81**
**ALL-IDB 2**					
mSFT	54.92 ± 6.64	52.96 ± 4.45	**84.62 ± 13.62**	25.23 ± 9.87	13.82 ± 15.65
mDFT	**58.38 ± 3.09**	**56.95 ± 2.42**	70.00 ± 12.38	**46.77 ± 11.66**	**17.60 ± 6.40**

**Table 6:** Results obtained using the Xception architecture according to 50 epochs (best values in bold).

Approach	A (%)	P (%)	R (%)	S (%)	MCC (%)
**ALL-IDB 1**					
mSFT	72.59 ± 6.76	**84.48 ± 12.96**	53.06 ± 20.10	**88.81 ± 12.26**	47.07 ± 11.54
mDFT	**77.41 ± 8.65**	69.40 ± 9.76	**94.69 ± 3.10**	63.05 ± 18.11	**60.33 ± 12.01**
**ALL-IDB 2**					
mSFT	59.31 ± 6.11	58.59 ± 5.89	71.85 ± 13.26	46.77 ± 23.12	18.94 ± 12.02
mDFT	**64.92 ± 2.60**	**63.58 ± 2.54**	**70.31 ± 7.06**	**59.54 ± 6.45**	**30.21 ± 5.25**

**Table 7:** Results obtained using ResNet50 according to 50 epochs (best values in bold).

Approach	A (%)	P (%)	R (%)	S (%)	MCC (%)
**ALL-IDB 1**					
mSFT	52.78 ± 4.14	9.07 ± 20.29	20.00 ± 44.72	80.00 ± 44.72	0
mDFT	**87.96 ± 2.70**	**91.59 ± 8.56**	**82.04 ± 4.42**	**92.88 ± 8.08**	**76.39 ± 5.50**
**ALL-IDB 2**					
mSFT	46.08 ± 8.77	16.05 ± 23.06	26.00 ± 43.36	66.15 ± 47.61	−7.84 ± 17.54
mDFT	**69.46 ± 6.26**	**66.96 ± 7.05**	**80.31 ± 10.17**	**58.62 ± 16.71**	**40.65 ± 12.17**

**Table 8:** Running time analysis for training (in minutes) and classification of an image (in seconds).

Model	Training Time (Min)	Classification Time (Image/s)
**ALL-IDB 1**		
VGG-16	34:43	0.0050
VGG-19	38:08	0.0055
InceptionV3	36:03	0.0111
Xception	40:26	0.0101
ResNet	38:08	0.0166
**ALL-IDB 2**		
VGG-16	35:28	0.0038
VGG-19	34:45	0.0041
InceptionV3	31:96	0.0065
Xception	38:00	0.0057
ResNet	37:05	0.0083

**Table 9:** Results obtained using different dimensionalities for the LeukNet’s fully connected layers (best values in bold).

Fc Layers	A (%)	P (%)	R (%)	S (%)	MCC (%)
**ALL-IDB 1**					
512–256	94.81 ± 2.41	93.92 ± 2.50	94.69 ± 3.09	94.91 ± 2.07	89.55 ± 4.88
1024–256	**97.04 ± 1.21**	**96.42 ± 2.45**	**97.14 ± 1.83**	**96.95 ± 2.21**	**94.07 ± 2.36**
1024–512	93.14 ± 3.73	92.95 ± 7.62	92.65 ± 3.09	93.55 ± 7.89	86.57 ± 6.85
1024–1024	93.70 ± 1.65	91.79 ± 3.11	94.69 ± 1.82	92.88 ± 3.03	87.43 ± 3.18
**ALL-IDB 2**					
512–256	71.53 ± 4.97	70.91 ± 4.20	74 ± 13.98	69.07 ± 9.90	43.95 ± 9.90
1024–256	**82.46 ± 0.02**	**77.59 ± 0.04**	**92.30 ± 0.08**	**72.61 ± 0.09**	**66.96 ± 4.96**
1024–512	71.84 ± 3.64	77.84 ± 12.16	67.53 ± 17.09	76.15 ± 23.46	47.17 ± 6.66
1024–1024	69.15 ± 2.11	69.91 ± 4.94	69.23 ± 9.41	69.07 ± 11.52	38.93 ± 3.73

**Table 10:** Feature space analysis performed with VGG-16 architecture as descriptor and a Linear SVM as classifier (best values in bold).

		*LODOCV*				*k*-Fold			
**Approach**	**Num. of Features**	**A (%)**	**P (%)**	**R (%)**	**Kappa**	**A (%)**	**P (%)**	**R (%)**	**Kappa**
**ALL-IDB 1**									
SFT	100	63.88%	56.75%	85.71%	0.3017	98.14%	96.07%	100%	0.9627
mSFT	256	75.00%	100%	44.89%	0.4709	96.29%	100%	91.83%	0.9247
DFT	100	59.25%	52.68%	100%	0.2362	**99.07%**	98%	100%	**0.9813**
mDFT	256	**87.96%**	86.00%	87.75%	**0.7575**	91.66%	95.45%	83.04%	0.8571
ImageNet	100	68.51%	59.49%	95.91%	0.3962	97.22%	96%	97.95%	0.9440
**ALL-IDB 2**									
SFT	100	60.38%	58.18%	73.84%	0.2076	88.07%	86.66%	90%	0.7615
mSFT	256	55.38%	54.54%	64.61%	0.1076	76.15%	77.86%	73.07%	0.523
DFT	100	51.92%	51.06%	92.30%	0.0384	**94.23%**	93.89%	94.61%	**0.8846**
mDFT	256	**73.84%**	75.83%	70.00%	**0.4769**	85.00%	84.21%	86.15%	0.7
ImageNet	100	49.61%	49.68%	60%	−0.007	87.69%	87.69%	87.69%	0.7538

**Table 11:** Comparison between the results obtained by the proposed method and the ones obtained by state-of-the-art methods (best values in bold).

Work	Number of Images	Validation Technique	A(%)
**Handcrafted Features**			
Putzu et al. [6]	267	*k*-fold	93.63
Vincent et al. [7]	100	holdout	97.70
Patel e Mishra [8]	27	holdout	93.75
Singhal et al. [9]	260	*k*-fold	93.80
**Deep-Learning-based systems**			
Thanh et al. [14]	1188	holdout	96.60
Shafique et al. [16]	760	holdout	99.50
Rehman et al. [17]	330	holdout	97.78
Loey et al. [18]	564	holdout	100
Pansombut et al. [19]	363	holdout	81.74
Ahmed et al. [20]	903	*k*-fold	88.25
**Feature extraction with CNNs**			
Vogado et al. [22]	1268	*k*-fold	**99.76**
Sahlol et al. [23]	260 and 10.921	*k*-fold	96.11 and 87.90
**LeukNet**	3536	*k*-fold	**98.61**

**Table 12:** Comparison between the proposed method (LeukNet) and the method suggested by Vogado et al. [22] with *k*-fold cross-validation in all 3415 images of the used 17 datasets.

Approach	Accuracy (%)	Precision (%)	Recall (%)	Specificity (%)	MCC (%)
Vogado et al. [22]	92.79	92.90	92.80	92.22	-
VGG-16 *	98.64 ± 0.43	98.66 ± 0.36	99.19 ± 0.50	98.02 ± 0.54	97.34 ± 0.008
LeukNet	98.61 ± 0.53	98.69 ± 0.47	99.24 ± 0.44	98.07 ± 0.69	97.45 ± 0.009

* Without transfer learning.

**Table 13:** Comparison of the proposed model with the method suggested by Vogado et al.  [22] by Leave-one-dataset-out cross validation in the UFG dataset (best values in bold).

Approach	Accuracy (%)	Precision (%)	Recall (%)	Specificity (%)	MCC (%)
Vogado et al. [22]	52.06	49.90	52.10	47.70	-
VGG-16 *	65.94 ± 6.85	66.06 ± 7.26	79.37 ± 12.90	50.87 ± 26.40	32.12 ± 13.11
LeukNet	**70.24** ± 5.51	**70.54** ± 8.62	**80.31** ± 15.17	**58.94** ± 22.24	**42.32** ± 10.31

* Without transfer learning.

## Data Availability

The data presented in this study is available at: https://git.io/JOCYu (accessed on 1 March 2021).
